# Leveraging a Partnership to Disseminate and Implement What Works in Family Planning and Reproductive Health: The Implementing Best Practices (IBP) Initiative

**DOI:** 10.9745/GHSP-D-18-00236

**Published:** 2019-03-22

**Authors:** Nandita Thatte, Asa Cuzin-Kihl, Ados Velez May, Margaret D'Adamo, Gifty Addico, James Kiarie, Ian Askew

**Affiliations:** a Implementing Best Practices Initiative, World Health Organization, Geneva, Switzerland.; b Implementing Best Practices Initiative, STAR Fellow, Washington, DC, USA.; c United States Agency for International Development, Washington, DC, USA. Now an independent consultant, Baltimore, MD, USA.; d United Nations Population Fund, New York, NY, USA.; e World Health Organization, Geneva, Switzerland.

## Abstract

The IBP initiative, a WHO-based partnership of NGOs, civil society organizations, governments, academic institutions, and other implementing partners, promotes evidence-based global guidelines, tools, and other interventions for local application, and incorporates implementation experience and learning back into the global discourse.

## BACKGROUND

The global health community has mobilized around various initiatives to advance the 2030 Sustainable Development Goals (SDGs) to improve the lives of women and children. The United Nations (UN) *Every Woman Every Child* strategy,^1^ Family Planning 2020 (FP2020),^2^ and most recently the World Health Organization's (WHO's) *Thirteenth General Programme of Work 2019–2023* (GPW13)^3^ have all emphasized the need for regional- and country-level investments, collaboration, partnership, and accountability to move the agenda forward.

But what does strong collaboration and partnership really look like? The Implementing Best Practices (IBP) initiative is an example of a longtime partnership dedicated to supporting the dissemination and use of evidence-based family planning and reproductive health guidelines, tools, and practices.^4^ IBP was created in 1999 with support from WHO, the United States Agency for International Development (USAID), and the United Nations Population Fund (UNFPA) to “effectively exchange and transfer knowledge, information, expertise and experience in order to improve practice.”^5^ Through its WHO-based Secretariat and growing network of more than 60 member organizations, spanning global, regional, and local NGOs and civil society organizations (CSOs), IBP has made significant efforts to bridge the gap between knowledge generated by the family planning community and the use of that knowledge to improve family planning and reproductive health outcomes. In addition, IBP has helped create a platform for field-based implementers to feedback local implementation experience and learning into the global discourse.

IBP has made significant efforts to bridge the gap between knowledge generation and knowledge use in family planning and reproductive health.

Over the past 15 years, IBP and its network of partners have made notable contributions to global family planning events and initiatives, such as the biennial International Conference on Family Planning^6^ and the Family Planning High Impact Practices (HIPs) collaboration,^7^ and have helped expand the global knowledge base on implementation and scale-up. At regional levels, IBP has used its leadership role to mobilize partners to support the East, Central and Southern Africa (ECSA)^8^ Best Practices Forums and the West African Health Organization (WAHO) Forums on Best Practices in Health.^9^ Recently IBP partnered with FP2020 and their regional focal points in Asia to host an IBP Asia Regional Workshop in Delhi, sparking new membership and revitalizing efforts for family planning knowledge exchange in the region. At the country level, IBP partners have convened workshops and conducted various activities such as documentation exercises and share fairs to support the dissemination and use of evidence-based interventions.

As family planning and reproductive health continues to gain momentum, we present IBP's strategic plan, explore some achievements of the initiative, and offer insights into how partners can leverage IBP to help achieve global development goals. More analytical information on how the collaboration has worked and areas for improvement is forthcoming, based on results of a midterm evaluation.

## IBP'S ROLE WITHIN GLOBAL REPRODUCTIVE HEALTH PARTNERSHIPS

Since the time IBP was started, the family planning and reproductive health landscape has evolved with new partners, donors, and alliances working to increase the visibility of family planning and international and national commitments to scale up family planning services. In 2004, the Reproductive Health Supplies Coalition was started, bringing together a diversity of partners to increase access to a full range of affordable, quality reproductive health supplies in low- and middle-income countries.^10^ Several years later, in 2011, the Ouagadougou Partnership committed to focusing efforts to increase access to voluntary family planning in 8 francophone countries in West Africa.^11^ In 2012 FP2020 was launched and has since mobilized countries and governments to provide 120 million more women and girls access to voluntary family planning by 2020.^12^ Most recently, a new financing mechanism, the Global Financing Facility (GFF), has also included family planning and reproductive health as part of its mandate to better finance the global strategy for *Every Woman Every Child*.^13^ All the while, existing stakeholders like UN agencies, country governments, donor agencies, academic institutions, NGOs, and CSOs continue to invest in efforts to strengthen and improve family planning and reproductive health outcomes. Each of these partnerships plays a unique role in advancing family planning advocacy, policy, financing, and programming globally.

The Figure illustrates some of the many stakeholders needed to ensure that global family planning goals are met. These stakeholders range from those focused on developing evidence-based guidelines and tools, to those galvanizing commitment and advocacy for an enabling environment, to those working on the ground to support quality implementation and scale up. The latter is where IBP has an important role to play as it is a platform dedicated to support the implementation and scale-up of evidence-based guidelines, tools, and practices.

**FIGURE:**
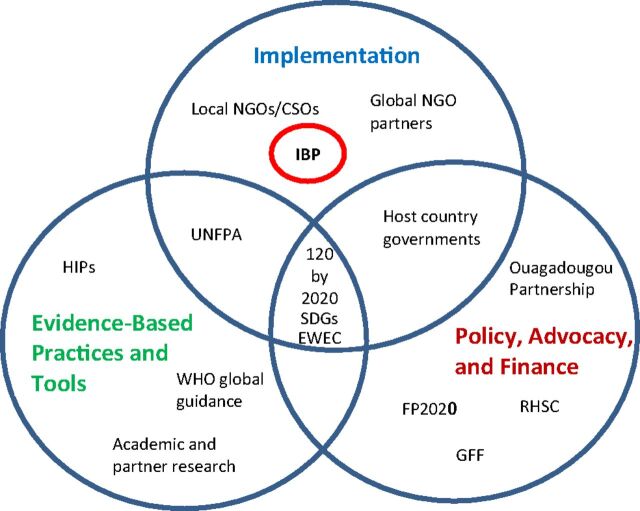
IBP in the Context of Other Development Actors in Family Planning Abbreviations: CSOs, civil society organizations; EWEC, *Every Woman Every Child*; FP2020, Family Planning 2020; GFF, Global Financing Facility; HIPs, Family Planning High Impact Practices; IBP, Implementing Best Practices; RHSC, Reproductive Health Supplies Coalition; SDGs, Sustainable Development Goals; UN, United Nations; UNFPA, United Nations Population Fund; WHO, World Health Organization. Note: “120 by 2020” refers to the FP2020 global mandate to reach 120 million more women and girls with voluntary contraception by 2020.

## IBP STRATEGIC PLAN 2016–2020

Previous assessments of IBP have documented its achievements and provided guidance to the current 2016–2020 IBP strategy.^14^^,^^15^ The Table outlines IBP's 2016–2020 strategic objectives along with some cross-cutting mechanisms used to achieve them.

**TABLE.** WHO/IBP Strategic Objectives 2016–2020

Objective	Mechanisms to Achieve the Objective
**Objective 1:** Increase access to evidence-based guidelines, tools and resources	Online knowledge management platform Technical webinars Partner dissemination
**Objective 2:** Support implementation and scale up	Communities of practice Development of tools Documentation
**Objective 3:** Enhance collaboration through increased partnership and coordination	Global and regional IBP meetings Engagement of new partners Linkages with other global networks
**Cross-cutting themes:** Institutionalization; Documentation; Monitoring and evaluation	

Abbreviations: IBP, Implementing Best Practices; WHO, World Health Organization.

### Objective 1: Increase Access to Evidence-Based Guidelines, Tools, and Resources

The family planning and reproductive health community has invested in a plethora of tools and approaches to further strengthen the provision of family planning services. The IBP strategy has focused on a selected set of tools for its core work, including WHO's *Medical Eligibility Criteria for Contraceptive Use* (MEC),^16^ *Selected Practice Recommendations for Contraceptive Use* (SPR),^17^ *Family Planning: A Global Handbook for Providers*,^18^ the *Training Resource Package for Family Planning*,^19^ and *Programming Strategies for Postpartum Family Planning*.^20^ In addition, IBP has increased its support of the HIPs, a set of evidence-based programmatic interventions in family planning. IBP has introduced each of these guidelines, resources, and practices to broader audiences through its webinars, its regional and country-based meetings, and its participation in periodic global and regional family planning meetings, forums, and events. In addition, IBP uses an online platform to communicate with members and facilitate learning about implementation and scale-up. This online platform enables IBP members to interact with each other on a low-bandwidth platform through an email-based interface. The platform hosts listservs and online communities of practice and facilitates knowledge exchange through discussion forums, shared libraries, and community calendars. IBPs webinar series also help to disseminate resources in a way that showcase technical content but also highlight programmatic field-based application. With IBPs wide network webinars regularly register over 150 participants from countries all over the world.

### Objective 2: Support Implementation and Scale-Up

In addition to dissemination, IBP has invested efforts to support the implementation, use, and scale-up of evidence-based interventions. IBP has facilitated the development of online communities of practice that allow participants to share their experiences about implementing and scaling up various interventions. In some instances, IBP has served as a launch pad for these communities to grow into larger separately funded networks. For example, the Health Information and Publications Network (HIPNet) and the Inter-Agency Working Group for Reproductive Health in Crises (IAWG) started on the IBP Knowledge Gateway but have since been able to secure resources to create separate online presences. In addition, IBP has provided the ability to archive and maintain institutional knowledge around technical topics beyond the life of any one project. For example, the Family Planning and Immunization Integration Working Group started under the Maternal and Child Health Integrated Program (MCHIP) and has continued to be active beyond the life of the project.

IBP has also contributed to the development of tools to support implementation and scale-up. In 2013, the WHO *Guide to Fostering Change to Scale Up Effective Health Services*^21^ was developed by IBP partners to illustrate a comprehensive way to institute change to support scale-up of health interventions. In 2018, this guide, along with other documented approaches to scale-up such as WHO and ExpandNet's *Nine Steps for Developing a Scaling-Up Strategy*,^22^ was revisited and consolidated into the *WHO Concise Guide to Implementing and Scaling Up Family Planning Service Improvements*.^23^ Efforts to create tools to better link clinical guidelines and programmatic interventions are also underway to further support use.

### Objective 3: Enhance Collaboration Through Increased Partnership and Coordination

IBP has benefited from a diverse network of members both through member organizations formally joining the initiative and individuals subscribing to IBP's online communities of practice. This diverse membership has allowed for collaborations and partnerships among partners based globally and in the region. Through IBP, organizations have collaborated on sessions for the International Conference on Family Planning (ICFP). Starting with the first meeting in Uganda in 2009 to the most recent meeting in Rwanda, IBP partners have worked together to develop technical panels, posters, and interactive knowledge exchange sessions as part of this global conference. Often the most interactive sessions during the conference, the IBP track has become known to foster dialogue and sharing around implementation experiences in FP. IBP has fostered collaboration among partners through participation in IBP webinars, partner meetings, and in documenting implementation experiences.

### Cross-Cutting Themes

In addition to the 3 primary strategic objectives, IBP has prioritized 3 cross-cutting themes to better support its work: institutionalization, documentation, and monitoring and evaluation.

*Institutionalization* refers to IBP partners' joint ownership of IBP and commitment to support their own individual work by engaging with IBP and its activities. Often times with global networks, “ownership” is restricted to those directly supporting the network at the global or headquarters level. This may occur through identified working groups, focal points, or, in the case of IBP, organizational points of contact. In order to ensure IBP is a known resource at all levels of member organizations and not just at headquarters level, efforts are being made to engage more staff beyond the main points of contact and among staff in regional and country offices. Institutionalizing IBP also means recognizing the importance of country and regional buy-in. To this end, IBP works deliberately at regional and country levels to ensure that key evidence-based guidelines, tools, and practices are introduced, adopted, and adapted as needed in line with the epidemiological, social, and cultural context. IBP support to the WAHO Forums on Best Practices in Health highlights the impact of these efforts. From the first forum in 2015, the WAHO Forums have now evolved to one of the premier conferences in the region mobilizing ministers of health, partners, and policy makers.

IBP works deliberately at regional and country levels to ensure key evidence-based guidelines, tools, and practices are adopted and adapted as needed.

*Documentation* is an overlooked but important investment to help understand what works and doesn't work when implementing public health interventions. IBP, in collaboration with partners, has developed a tool to better document such efforts. In 2013, in partnership with WAHO and the WHO Regional Office for Africa, IBP supported the development of a documentation guide template. The template was further adapted by WAHO and the WHO Regional Office for Africa, and in 2017 was published as *A Guide to Identifying and Documenting Best Practices in Family Planning Programmes*.^24^ IBP partners have used this documentation guide to help document adolescent-friendly contraceptive services in Latin America and India and the provision of family planning in drug shops in Ghana. In addition, the guide was identified as a key resource as part of the 2018 WAHO Forum on Best Practices in Health and reached 1,600 downloads in the 6 months leading up to the forum. Documentation of implementation experiences can help us not only understand how and why evidence-based resources are being used but also ensure this ‘experiential evidence’ is fed back into the formal evidence base.

*Monitoring and evaluation* are critical to measure success and better document both successful and less successful experiences around implementation and scale-up. IBP has invested in monitoring and evaluation by conducting a baseline survey of the IBP 2016–2020 strategy assessing the use of IBP-supported resources. Preliminary results indicate that the tools used most by members were WHO's MEC, through use of the MEC wheel,^25^ and the HIP briefs.^7^ Use varied based on the type of resource. For example, the MEC wheel and the Training Resource Package were used for in-service provider training, whereas the HIP briefs and the WHO Postpartum Family Planning Compendium^26^ were used for advocacy and to inform policies. Over 50% of IBP member organizations reported limited time and resources as a major barrier to expanded use. Challenges linking clinical guidelines to programmatic needs were commonly reported barriers for use of WHO guidelines, whereas difficulty measuring impact and limited country contextualization were reported as barriers for using the HIP briefs. Despite some challenges in use of IBP-supported resources, a majority (95%) of IBP member organizations participated in at least some IBP activities, whether they were partner meetings or communities of practice, and over 85% reported their membership in IBP added value to their work. Efforts to conduct a midterm survey are underway that will incorporate more field-based insights.

## THE ADDED VALUE OF IBP

### A Neutral Platform That Reduces “Collabetition” and Promotes Collaboration

The IBP Secretariat is housed at WHO, which provides a neutral space for IBP members to exchange learning experiences, resources, tools, and lessons learned. IBP member organizations contribute their own funds to support joint IBP activities, which also prevents any competition for funds among IBP members. The term “collabetition” was coined by IBP partners to capture the challenge faced when trying to balance the pressure by donors and governments to collaborate in a time when there is competition for limited donor and government resources.^14^ Through its neutral convening platform, IBP has moved away from *collabetition* toward more *collaboration* between its partners. Collaboration among IBP member organizations is evident through the IBP tracks at the biennial International Conference on Family Planning, pre-conference workshops, and joint technical webinars and partner meetings. For example, IBP webinars regularly include more than 150 registrants from over 50 countries around the world. IBP's neutral platform also allows tools to be featured in, for example, Uganda's first family planning conference, held in 2014. At the time, political reasons prohibited U.S. government and U.S.-funded partners from attending. However, the neutrality of IBP and the important connection between IBP and WHO enabled IBP to attend, ensuring that evidence-based resources were shared as part of this important conference.

### Bringing Implementer Perspectives to Global Guidelines and Research

IBP can serve as a broker bringing implementation perspectives to create user-friendly derivative tools for global guidelines and to inform implementation research agendas. For example, in 2016 as WHO was developing an implementation guide to facilitate the incorporation of the MEC and SPR guidelines into national policies and programs,^27^ IBP convened a day-long workshop among its partners to provide feedback on the draft and solicit input on their use of the MEC wheel and SPR. Feedback revealed that while the MEC wheel and SPR guidelines were useful, the cost of the MEC wheel was prohibitive and the SPR guidelines document was bulky in nature and structured in a way that made it difficult to find relevant information. Feedback from IBP partners guided the preparation of a new decision support tool targeted for frontline providers of contraception in humanitarian settings. The tool presents key information from both the MEC and SPR in one place in a practical, simplified format and is available as an app for iOS and Android devices.^28^ More recently, IBP and the WHO Alliance for Health Policy and Systems Research (the Alliance) collaborated to develop a joint call for implementation research on service provision in drug shops. Prior to developing the research call, IBP helped leverage a technical working group meeting on drug shops and pharmacies to gather field-based experience from IBP implementers. Preliminary results highlighted both expected issues such as accreditation and supply chains and unexpected ones such as motivation of drug shop owners as barriers to service delivery. These topics added a different dimension to the research themes and were used to help inform the final research call.

### A Network Approach with Global, Regional, and Local Partners

Over the years, IBP has expanded its membership to more than 60 member organizations. In addition to member organizations, individual subscribers to IBP's online communities of practice have reached close to 80,000 in more than 150 countries. IBP member organizations include global NGOs, academic institutions, regional bodies, and CSOs. Individual members are equally diverse and represent governments, students, policy makers, researchers, and community activists among other groups. The breadth of the IBP network also enables IBP to reach local NGOs and CSO partners who may not be part of larger global initiatives or global projects. This network approach also helps to foster unique collaboration that may otherwise rarely occur. For example, in 2018, IBP partner organizations Equipop and the YP Foundation were introduced to each other at an IBP regional meeting in India. The YP Foundation was implementing youth audits for sexual and reproductive health services in local clinics to improve youth engagement and quality services. Equipop was hoping to implement a similar strategy in Burkina Faso. Rather than start from scratch, the YP Foundation made a video for Equipop showcasing their approach to youth-led audits. Equipop then translated the video into French and used it in their training on youth audits among the Burkina Faso team (personal communication, Manak Matiyani, YP Foundation and Elise Petitpas, Equipop). This example of a collaboration between a youth-led Indian NGO and a French-based NGO working in West Africa showcases the unique advantage IBP has in bringing a range of global-, regional-, and country-level organizations together. Fostering interactive knowledge sharing and encouraging collaboration through hands-on facilitation, IBP creates opportunities for such collaboration to occur.

IBP has more than 60 member organizations and close to 80,000 individual subscribers to its online communities of practice.

## A WAY FORWARD AND GETTING INVOLVED

IBP has come a long way since its inception in 1999. A focus on disseminating key guidelines, tools, and resources, efforts to strengthen implementation, and a concerted effort to expand the partnership have yielded some exciting activities and unique collaboration opportunities. In order to further elevate the profile of IBP, additional efforts are underway to strengthen the initiative and engage more partners in its vision.

### Measuring Progress and Telling the IBP Story

IBP will continue to communicate its value to the family planning and reproductive health community at large through rigorous measurement and documentation of its work. Monitoring, evaluation, and documentation have been prioritized in the new 2016–2020 strategy, and surveys and in-depth interviews with key stakeholders are underway.

In addition to quantitatively assessing the use of tools and guidelines, IBP will also work to better capture the experiential and field-based stories from IBP partners who are using IBP-supported tools and resources in the field. IBP will also explore further the mechanisms of collaboration, partnership, and successful communities of practice. We encourage current IBP member organizations to help document the added-value of the IBP network through blog posts, articles, or social media posts illustrating collaborative efforts, knowledge exchanges, or connections made as a result of IBP activities.

### Expanding Membership to More Local and Regional Partners

IBP will continue to expand its membership, with a focus on engaging more regional and local partners from Africa, Asia, Europe, and Latin America and strengthening efforts to support more regional- and country-level workshops and activities. Since 2017, IBP has welcomed more than 16 new member organizations ranging from global partners such as International Medical Corps to organizations in the European Region like Share-Net International, to local partners such as the Love Matters in India. IBP is well-placed to support better engagement with CSOs through these local and regional opportunities. Leveraging local and regional partners also helps align IBP with the WHO GPW13 that encourages new partnerships with local and regional organizations in order to show impact at country level. We encourage organizations to consider joining the IBP network or, at minimum, participating in IBP activities. Membership is simple and provides a unique opportunity to stay connected not only with the range of partners in the network but also with global partners such as UNFPA, WHO, and USAID.

### Strengthening Coordination with Other Family Planning and Reproductive Health Efforts

As referenced earlier, there are many more partnerships, donors, and organizations dedicated to family planning and reproductive health today than ever before. In order to fully maximize these resources, we should recognize and use the comparative advantages of each to its fullest potential. IBP has already made strong linkages with the HIPs initiative and has been supporting dissemination of HIPs through global webinars and regional activities and has developed tools to better facilitate the use of HIPs. IBP will continue to strengthen coordination with FP2020, particularly with the FP2020 civil society focal points. IBP has long been a clear link between implementing partners and WHO, and IBP will continue to leverage this relationship with WHO Headquarters as well as WHO regional and country offices. As IBP grows, opportunities to further explore linkages with partnerships such as the Partnership for Maternal, Newborn and Child Health and the GFF will be prioritized to complement efforts around family planning and reproductive health.

## CONCLUSION

The diversity of IBP member organizations has enabled wide-ranging collaboration between other global and regional networks and organizations from around the world. As more organizations, donors, and partners invest in family planning and reproductive health efforts, it is important for IBP to maintain its comparative advantage as a consistent and committed partnership dedicated to promoting knowledge exchange around implementation and scale-up of evidence-based guidelines, tools, and practices in family planning and reproductive health. Investments to better understand and measure successful collaboration through IBP will help shape the way new partnerships and other collaborative efforts are developed. As the global health community rallies around FP2020, *Every Woman Every Child*, and the SDGs, capitalizing on the strengths of partnerships like IBP will be an important step to help us achieve the ambitious goals to improve the lives of women, children, and families worldwide.

## Notes

**Peer Reviewed**
